# Absenteeism-disease in health care workers in a hospital context in southern Brazil

**DOI:** 10.47626/1679-4435-2020-521

**Published:** 2021-03-03

**Authors:** Larissa Garcia de Paiva, Graziele de Lima Dalmolin, Wendel Mombaque dos Santos

**Affiliations:** 1 Programa de Pós-graduação em Enfermagem, Universidade Federal de Santa Maria, Santa Maria, RS, Brazil; 2 Serviço de Saúde Ocupacional e Segurança do Trabalhador, Hospital Universitário de Santa Maria, Empresa Brasileira de Serviços Hospitalares, Santa Maria, RS, Brazil

**Keywords:** occupational health, absenteeism, health care workers, hospital

## Abstract

**Introduction:**

Absenteeism is a pervasive and growing worldwide problem. In hospital settings, it is often associated with a fast-paced environment, shift work and high occupational demands. Absenteeism in hospitals can also be attributed to poor working conditions and the high emotional burden associated with daily exposure to illness and death. These conditions often lead to sickness absence.

**Objectives:**

To assess sickness absence among health care workers in hospital settings.

**Methods:**

A cross sectional study was conducted using the data and medical records of health care workers in a hospital in Rio Grande do Sul, Brazil. The data covered the period of September 2014 to December 2018.

**Results:**

The sample consisted of 559 workers, 233 of whom were absent for at least 1 day in the year. Sickness absence was most common among women (79%) and nursing technicians (45.5%). The mean duration of absence was 5.53 days (standard deviation: 20.42), and the sickness absence rate was 2.01%. The most common reasons for sickness absence were injury, poisoning and other consequences of external causes (20.19%), followed by mental and behavioral disorders (17.90%) and diseases of the musculoskeletal system and connective tissue (11.69%).

**Conclusions:**

Greater awareness of the factors associated with sickness absence in hospital settings can contribute to the planning of occupational health initiatives targeting the most vulnerable workers.

## INTRODUCTION

Absenteeism refers to the failure to report to work due to personal issues or illness for 1 or more expected work days (or shifts).^1-3^ It is a pervasive and growing worldwide problem, and interferes with productivity, service quality and the workload of other employees.^1,2^ Absenteeism is associated with extra costs, discontinuity of service, loss of productivity and other economic effects that result in increased expenditure.^1-3^

The increased job demands and workload affect the health of workers, leading to changes in their morbidity and mortality rates.^1,2,4^ Occupational hazards such as silicosis, asbestosis and fatal occupational injuries still persist in more precarious work environments. However, there has also been an increase in the prevalence of mental and behavioral illnesses, which account for an increasing number of absences, especially in younger workers.^4^

In the health care sector, these issues affect the workers themselves, but also the patients under their care. The public and private health care sectors in Brazil employ approximately 1.6 million people, who work in different environments with varying degrees of exposure to biological, physical and/or psychological risks or hazards.^3,5,6^

Health care work is fast-paced, shift-based and highly demanding; it is often associated with poor working conditions and a high emotional burden due to daily exposure to illness and death.^7^ In addition to the psychological burden and pressures of productivity and job demands, health care workers are exposed to several risk factors that may aggravate preexisting conditions and/or occupational injuries.^1,2,7^ Therefore, the workers’ relationship to their job, combined with the high demands for productivity, exert an influence that goes beyond the work environment and relationships, and ultimately affects both personal and health-related factors.^7,8^

A literature search was performed in the PubMed, Scopus and Web of Science databases in July 2019, for studies published since the proposal of the Global Plan of Action for Workers’ Health (2008). The search was performed using the following keywords: 1) Absenteeism: “Absenteeism”, “Sick leave”, “Absentee”; 2) Health care workers: “Health Personnel”, “Health Manpower”, “Healthcare Workers” and 3) Hospital Environment: “Hospital”, combined with different Boolean operators. A total of 1022 articles were retrieved, few of which discussed absenteeism in federal public servants working in tertiary health care centers.^4,5,7-9^ Studies of federal public servants in other sectors, however, found that the main causes of absenteeism were mental, behavioral and musculoskeletal disorders.^5,10-15^

Given the numerous risk factors and conditions that affect the health of workers and their ability to work, the aim of this study was to describe the pattern of absenteeism and identify the rates of sickness absence and associated factors in the first year of work among hospital employees.

## METHODS

This was a cross-sectional study based on data from the occupational health service of a hospital in Rio Grande do Sul, Brazil. Data were collected on sociodemographic, occupational (occupation, working hours) and individual variables (gender, age, occupation, working hours, medical history), as well as sickness-absenteeism (days of absence, illnesses according to the International Classification of Disease [ICD-10]).

The study was conducted in a tertiary teaching hospital where all patients were referred from the Unified Health System (SUS). The institution served a population of 1.2 million people across 35 cities. Nearly 6,000 people pass through the hospital every day, including patients and relatives, students, workers, residents and professors.

In December 2013, the hospital signed a contract with the Brazilian Hospital Services Company (EBSERH), which led to changes in local structures and management as they adapted to the new administration. New public service workers were hired starting in September 2014, which was selected as the start of the study period.

### POPULATION

The population consisted of health care workers with employment contracts based on the Consolidated Labor Laws (CLT). According to a report by the local occupational health service which listed the number of workers per area and sector from 2014 to 2018, the total population of this study consisted of 559 workers.

### INCLUSION AND EXCLUSION CRITERIA

All workers admitted on or after September 2014 who had been at the hospital for at least 1 year were included in this study. Health care workers transferred to or from other hospitals; on maternity leave; with no periodic medical examinations; and/or who had delayed these exams were excluded from the study.

### DATA COLLECTION

Data were collected in October 2018 from a database maintained by the hospital’s Occupational Health Service. Information was obtained from the records of pre-recruitment medical examinations, periodic assessments and other medical appointments related to work absences, all of which were in an Excel spreadsheet containing data from September 2014 to December 2018.

The following variables were investigated: occupation, gender, type and duration of leave taken, comorbidities, medical records with ICD-10 codes and occupational assessments undertaken at the hospital.

Data were extracted from the routine database of the hospital’s occupational health department to an electronic spreadsheet using Microsoft Excel 365.

### DATA ANALYSIS

Population characteristics were summarized using descriptive statistics. Categorical variables were described using frequencies and percentages, while quantitative variables were summarized using measures of central tendency (mean, median) and dispersion (standard deviation [SD], interquartile range), depending on the normality of the distribution, which was assessed using the Kolmogorov-Smirnov test. Analyses were then conducted using PASW Statistics (Predictive Analytics Software, SPSS Inc., Chicago, EUA), version 21.0.

Sickness absence rates were calculated according to the following formula, based on recommendations from the Sub-Committee on Absenteeism of the International Association of Occupational Health (ICOH, 1973):

**Table:** 

Sickness absence rate = number of absent days in 1 year × 100 number of possible working days^*^

* Depending on weekly hours and occupation.

Chart 1 shows the number of annual working days for each occupation.

**Chart 1:** Number of working days in the first year on the job for each occupation, Rio Grande do Sul, 2018

Occupation	Working days
Biomedical scientist	255
Nursing technician	307
Radiology technician	209
Health technician	255
Occupational therapist	255
Dentist	255
Nurse	307
Pharmacist	255
Physical therapist	255
Speech pathologist	255
Physician	157
Nutritionist	255
Psychologist	255

The risk of absenteeism was calculated using 2 × 2 tables to identify the absolute and relative risks associated with each of the characteristics examined.

### ETHICAL CONCERNS

All procedures were conducted according to the ethical guidelines of Resolution 466/2012. The present study was approved by the local research ethics committee (REC) under number CAAE: 80587417.0.0000.5346 and report No. 2.969.629. The researchers signed a data use agreement affirming their commitment to ensuring the confidentiality, privacy and security of the data. The institution also provided written authorization for researchers to access the data. Since this was a retrospective study of an institutional database, it did not require informed consent.

## RESULTS

The sample consisted of 559 health care workers, 41.7% (n = 233) of whom had been absent for at least one day in their first year of work. A total of 79% (n = 441) of participants were female; 45.5% (n = 254) were nursing technicians; and 71.2% (n = 398) worked 36 hours a week. The mean number of days off for these workers in 1 year was 5.53 (SD = 20.42). The median showed no days off, with an interquartile interval of no days (25%) and 3 days (75%).

Questions regarding familial risk factors revealed that 26.6% of participants had a family history of diabetes; 18.7% had relatives with hypertension; 35.3% had family members with heart disease; 4% had relatives with depression; 16.5% had a family history of vascular disease and 32.3% had family members with cancer. Only 4.2% of the sample reported previous occupational accidents, and no participants had a history of repetitive strain injury or work-related musculoskeletal disorders.

On items regarding medical history, illnesses and lifestyle, 64.1% of participants reported frequent physical activity, 22.7% consumed alcohol, 8% were obese and 8% had rhinitis (Table 1).

**Table 1:** Medical history, illnesses and lifestyle of hospital workers, Rio Grande do Sul, 2018

Lifestyle, illnesses and medical history	Yes	No
	n (%)	n (%)
Physical activity	319 (64.1)	240 (35.9)
Alcohol intake	127 (22.7)	432 (77.3)
Obesity	40 (8.0)	519 (92.0)
Rhinitis	40 (8.0)	519 (92.0)
Headaches	33 (6.6)	526 (93.4)
Rheumatic disease	30 (6.0)	529 (94.0)
Medication use	24 (4.8)	535 (95.2)
Back pain	18 (3.6)	541 (96.4)
Systemic arterial hypertension	17 (3.4)	542 (96.6)
Joint pain	16 (3.2)	543 (96.8)
Allergies	15 (3.0)	544 (97.0)
Smoking	15 (2.7)	544 (97.3)
Sinusitis	12 (2.4)	547 (97.6)
Depression	12 (2.4)	547 (97.6)
Indigestion	9 (1.8)	550 (98.2)
Cough	8 (1.6)	551 (98.4)
Anxiety	7 (1.3)	552 (98.7)
Stomach pain	6 (1.2)	553 (98.8)
Insomnia	5 (1.0)	554 (99.0)
Asthma	5 (1.0)	554 (99.0)
Diabetes	5 (0.9)	554 (99.1)
Tingling	3 (0.6)	556 (99.4)
Nervousness	2 (0.4)	557 (99.6)
Hypothyroidism	2 (0.4)	557 (99.6)
Constipation	1 (0.2)	558 (99.8)
Hemorrhoids	1 (0.2)	558 (99.8)
Hyperthyroidism	1 (0.2)	558 (99.8)

The analysis of absences in the first year of work showed that most participants did not miss any work days during this period (Table 2). Workers in the present study missed 2.01% of possible workdays due to sickness (sickness absence rate).

**Table 2:** Number of absent days among hospital workers over the course of 1 year, Rio Grande do Sul, 2018

Absent days	First year on the job n (%)
0	326 (58.3)
1-5 days	137 (24.5)
6-14	44 (7.9)
15-30	31 (5.5)
31 or more	21 (3.8)
Total	559 (100.0)

Table 3 describes the ICD-10 codes for the illnesses or reasons for work absence provided by participants. Mental and behavioral disorders accounted for the largest share of absent days (47.5%), followed by injury, poisoning and other consequences of external causes (40.9%), as well as diseases of the musculoskeletal system and connective tissue (22.4 days). The most common reasons for sickness absence were injury, poisoning and other consequences of external causes (20.2%), followed by mental and behavioral disorders (17.8%) and diseases of the musculoskeletal system and connective tissue (11.7%).

**Table 3:** Distribution of sickness absence days according to the International Classification of Diseases (ICD)-10, Rio Grande do Sul, 2018

Illness (ICD-10)	Mean days per leave	Total days n (%)
S00-T98 - Injury, poisoning and certain other consequences of external causes	40.9	696 (20.2)
F00-F99 - Mental and behavioral disorders	47.5	617 (17.8)
M00-M99 - Diseases of the musculoskeletal system and connective tissue	22.4	403 (11.7)
Z00-Z99 - Factors influencing health status and contact with health services	13.0	391 (11.3)
A00-B99 - Certain infectious and parasitic diseases	16.6	299 (8.7)
J00-J99 - Diseases of the respiratory system	9.4	198 (5.7)
K00-K93 - Diseases of the digestive system	7.6	167 (4.8)
J00-J99 - Diseases of the genitourinary system	13.3	159 (4.6)
O00-O99 - Pregnancy, childbirth and the puerperium	16.9	118 (3.4)
I00-I99 - Diseases of the circulatory system	9.7	97 (2.8)
H60-H95 - Diseases of the ear and mastoid process	7.8	78 (2.3)
L00-L99 - Diseases of the skin and subcutaneous tissue	7.0	63 (1.8)
E00-E90 - Endocrine, nutritional and metabolic diseases	7.6	53 (1.5)
H00-H59 - Diseases of the eye and adnexa	4.3	51 (1.5)
R00-R99 - Symptoms, signs and abnormal clinical and laboratory findings, not elsewhere classified	2.5	30 (0.9)
C00-D48 - Neoplasms	2.3	16 (0.5)
G00-G99 - Diseases of the nervous system	1	10 (0.3)
V01-Y98 - External causes of morbidity and mortality	1	1 (0.1)
Q00-Q99 - Congenital malformations, deformations and chromosomal abnormalities	0	0 (0.0)

The association between absenteeism and gender, age, occupation, weekly hours, medical history, illnesses and lifestyle was also analyzed. Only three of these variables were significantly associated (p < 0.05) with sickness absence: gender, weekly hours and smoking (Table 4).

**Table 4:** Variables associated with sickness absence among health care workers, Rio Grande do Sul, 2018

Variable	Noabsences		Absences		Absolute risk^*^	Relative risk (95%CI)
	n	%	n	%		
Gender						
Male	127	72.2	49	27.8	48 per 100	1
Female	199	52.0	184	48.0	89 per 100	1.86 (1.44-2.41)
Smoking						
No	282	58.4	201	41.6	42 per 100	1
Yes	3	20.0	12	80.0	81 per 100	1.92 (1.46-2.52)
Weeklywork hours						
24	113	78.5	31	21.5	42 per 100	1
30	22	61.1	14	38.9	63 per 100	2.32 (1.06 - 5.05)
36	170	50.6	166	49.4	73 per 100	3.59 (2.26-5.58)
40	21	48.8	22	51.2	74 per 100	3.81 (1.86-7.82)

95%CI = 95% confidence interval.

* Number of employees with at least 1 absent day per 100 workers.

Further analyses showed that women were 1.86 times more likely to have been absent (95% confidence interval [95%CI], 1.44 - 2.4) relative to men. Smokers were also 1.92 times more likely (95%CI, 1.45-2.52) to have been absent than non-smokers. Lastly, the comparison of participants with different work hours revealed that individuals who worked 40 hours a week were 3.81 times more likely than their peers to be absent at some point in the year (Table 4).

## DISCUSSION

The sickness absence rate in the present study was approximately 2%, which is in line with the value estimated by the International Labor Organization, which is the equivalent of 2.5%.^16^ The absence rates calculated by national and international studies range from 3 to 9.7% depending on the occupation and the characteristics of the health care service.^7,8,10-12^

The unwillingness to miss work shortly after starting a job, even in case of illness, as well as the short exposure time and the motivation to demonstrate productivity in a competitive environment may have contributed to the low absence rates in the present study, especially in recently hired workers, who may be serving a probationary or trial period and be afraid of losing their jobs.^17,18^

Approximately 41% of workers were absent for at least 1 day in their first year of work. Most absence spells (24.5%) lasted less than 5 days. The short duration of these periods may be attributed to the high frequency of single-day leaves for medical appointments and the short recovery time of the acute illnesses experienced by workers.^3,6,7,10,15,18-20^

In this study, the most prevalent causes of sickness absence, which accounted for approximately 50% of cases, were external causes, followed by mental and behavioral disorders and musculoskeletal issues. The literature suggests that most absences in health care sectors are due to mental and behavioral disorders, or musculoskeletal issues; however, unlike the present study, previous investigations have found these conditions to be associated with longer absence periods.^1,7,8,10-12,15,18,19,21^

Externally caused injuries consist of a diverse set of trauma-related pathologies, including poisonings and burn injuries, which can be related to workers’ lifestyle and health habits. In some cases, work absence can be attributed to the gradual onset of illnesses, and may be associated with gender, age, place of work, workload, work hours, and general health status.^2,4,5,9,22^ In this study, absence rates were associated with gender, weekly working hours and overall health status, which includes smoking status.

Women were 1.86 times more likely to be absent than men; a similar discrepancy has also been observed in previous studies, and may be explained by the “second shift” phenomenon (child care and domestic activities) as well as the higher levels of stress and general health issues associated with female gender.^22-26^ Additionally, health care workers are predominantly female, especially in the field of nursing, where the workforce is larger and therefore accounts for a larger number of absences.^9,19,24,25^

Workers who smoke have nearly double the risk of sickness absence, and smoking in turn is often related to stress and anxiety.^27,28^ Smoking also increases the risk of respiratory and cardiovascular illnesses, in addition to cancer.^27,28^ The implementation of smoking cessation programs can therefore contribute to reduced absenteeism and improved quality of life in these workers.^27,29,30^

Increased work hours are also a risk factor for absenteeism. The number of workers with at least one absence increased from 21 to 31 and 32 per 100 workers among those working 30, 36 and 40 hours a week, respectively, relative to those with a 24-hour work week. The increase in absenteeism for those working longer hours is attributed to the high demands and workload experienced by these individuals, especially those working in hospital settings.^1,21-23,25,26^

Some inherent features of health care occupations, such as shift work; occupational overload; the need to carry out repetitive and physically demanding activities; high psychological burden; and a fast pace of work may contribute to illness onset, and have an even greater influence as work hours increase.^1,21-23,25,26^

## CONCLUSIONS

The sickness absence rate for health care workers in a hospital in southern Brazil was 2.01%. The median duration of absences was 4 days per worker. Female gender, smoking and a work week of 40 hours or more were associated with higher rates of sickness absence. The results also showed that the most common reasons for sickness absence were injury, poisoning and other consequences of external causes (20.2%), followed by mental and behavioral disorders (17.8%) and diseases of the musculoskeletal system and connective tissue (11.7%).

The identification of factors associated with higher sickness absence rates can contribute to the development and planning of prevention and promotion strategies in occupational health. Such strategies can improve the quality of life of workers, optimize work processes and increase the quality of health care. Information regarding illness and health factors in health care workers can also provide a basis for managerial decision making.

The acknowledgment and awareness of the reality of health care workers will allow for the implementation of early interventions to reduce sickness absence rates, while improving the services offered to these workers. The analysis of prevalent risk factors for sickness absence and the improved screening for health and sickness factors may allow for the development of more effective action plans to improve workers’ quality of life.

Internal actions with a direct impact on worker health, such as a greater focus on preventive actions, improved support services, the follow-up of workers with a history of illness and the reorganization of the work environment in order to adjust it to the needs of each employee could increase worker satisfaction and reduce absence rates.

The present results should be interpreted in light of a few limitations. Firstly, the data collected from pre-recruitment medical examinations may have been influenced by workers’ unwillingness to share personal information early in the employment relationship. Secondly, the data pertain to a specific population of hospital workers in the southern region of the country, which may not be representative of workers in other locations. Nevertheless, this study provides a basis for the identification of issues that are likely to affect hospital workers everywhere, and should be investigated in other settings across Brazil.
